# Construction Notes

**Published:** 1893-05-06

**Authors:** 


### CONSTRUCTION NOTES.

The hospital in connection with the East Poorhouse, Dundee, is being fitted throughout with heating steam cooking apparatus and laundry machinery by the firm of Harper Twelvetrees, engineers, London and Manchester.

Considerable alterations are to be effected in connection with the flooring and the heating apparatus of the Lincoln County Hospital. It is proposed to put double stoves in the place of single ones, and to refloor certain wards which greatly require repairs. The surveyor estimated the cost at about £800. A new disinfecting apparatus is to be added to the existing establishment.

St. Mary's Hospital, Manchester.—The erection of the new buildings for this hospital are about to be commenced, the final plans being now under consideration. The site of the buildings is in Oxford Street and Gloucester Street, which has cost £16,000, and they will accommodate 100 beds. Although some £40,300 has been subscribed to the building fund, there will still be required, the Board of Management state, a considerable sum to complete the work. Mr. Waterhouse, R A., is the architect.

A special meeting of the Birkenhead Town Council was held the second week in April for the purpose of considering a recommendation by the Health Committee to erect a new hospital for infectious diseases at Flaybrick Hill. The building is to contain an administrative block ; four double wards, to contain twelve beds each ward ; one isolation ward, to contain eight beds ; with laundry, disinfecting block, and mortuary. The total amount required to complete the hospital in all its details is about £20,000, including the land.

The rural sanitary authority of the Rochford Union have applied to the Local Government Board for sanction to borrow £2,100 for the erection of an infectious diseases hospital for that district. The construction of the proposed hospital will comprise two wards. It will be erected on two acres of land, enclosed with a fence six feet high, and there will be a gate for admittance, the fence being forty feet from the building. The building will be drained with six and nine inch pipes, with manholes and ventilating shafts. There will be five water-closets, connected to the sewer with six-inch pipes. There are also in contemplation two flushing cisterns. The linen is to be dried in a drying chamber.

Jewish Convalescent Home, Hove.—This Home has been erected at Hove as a seaside branch of the Norwood Jewish Convalescent Home. The accommodation consists of large dining-room, matron's room, men's sitting-room, women's sitting room, children's room, two bath rooms, w.c.'s, kitchen and domestic offices, and separate entrances for men and women on the ground floor. On the first floor : nine bedrooms and two lavatories, with two staircases, the men's bedrooms and lavatory being separated from the women's. Two bedrooms are situated in the roof. The Home is built of stock bricks, the ground-floor being faced externally with red bricks, and with an internal lining of hygienic rock, the walls of upper floor being faced with red hanging tiles, the roof also being covered with red tiles. Messrs. T. Lainson and Son are the architects.

Holbeck Nurses' Home.—A nurses' home at Holbeck, in connection with the Leeds Association for Nursing the Sick Poor, is in course of erection. A piece of vacant ground in Domestic Street, offering a suitable site, Mrs. Meynell-Ingram made a free gift of this land to the committee. The plans for the building were selected from designs presented by Mr. Walter A. Hobson, of Leeds, and the entire contract or the same was accepted from Mr. Barber at an estimated cost of about £850. The architect's designs show a double-fronted building of pleasing, simple design and appearance. The exterior elevation is faced with pressed bricks, and relieved with stone dressings, the roof being covered with dark Welsh slates. The ground floor comprises an entrance hall five feet wide, dwelling rooms, kitchen and scullery. The upper floor contains the bed-rooms and bath-room, the entire site covering about 400 square yards.

